# Electromyographic analysis of masseter muscle in newborns during suction in breast, bottle or cup feeding

**DOI:** 10.1186/1471-2393-14-154

**Published:** 2014-05-01

**Authors:** Ellia CL França, Cejana B Sousa, Lucas C Aragão, Luciane R Costa

**Affiliations:** 1 Health Sciences Graduate Program, Federal University of Goias, Goiania, GO, Brazil; 2 Department of Speech, Language and Hearing Sciences, Pontifical Catholic University of Goiás, Goiania, GO, Brazil; 3 Department of Neuropsychiatry, Federal University of Pernambuco, Recife, PE, Brazil; 4 Division of Pediatric Dentistry, Faculty of Dentistry, Federal University of Goias, Goiania, GO, Brazil

**Keywords:** Electromyography, Masseter muscle, Newborns, Breastfeeding, Bottle feeding, Cup feeding

## Abstract

**Background:**

When breastfeeding is difficult or impossible during the neonatal period, an analysis of muscle activity can help determine the best method for substituting it to promote the child’s development. The aim of this study was to analyze the electrical activity of the masseter muscle using surface electromyography during suction in term newborns by comparing breastfeeding, bottle and cup feeding.

**Methods:**

An observational, cross-sectional analytical study was carried out on healthy, clinically stable term infants, assigned to receive either breast, or bottle or cup feeding. Setting was a Baby Friendly accredited hospital. Muscle activity was analyzed when each infant showed interest in sucking using surface electromyography. Root mean square averages (RMS) recorded in microvolts were transformed into percentages (normalization) of the reference value. The three groups were compared by ANOVA; the “stepwise” method of the multiple linear regression analysis tested the model which best defined the activity of the masseter muscle in the sample at a significance level of 5%.

**Results:**

Participants were 81 full term newborns (27 per group), from 2 to 28 days of life. RMS values were lower for bottle (mean 44.2%, SD 14.1) than breast feeding (mean 58.3%, SD 12.7) (*P* = 0.003, ANOVA); cup feeding (52.5%, SD 18.2%) was not significantly different (*P* > 0.05). For every gram of weight increase, RMS increased by 0.010 units.

**Conclusions:**

Masseter activity was significantly higher in breastfed newborns than in bottle-fed newborns, who presented the lowest RMS values. Levels of masseter activity during cup-feeding were between those of breast and bottle feeding, and did not significantly differ from either group. This study in healthy full term neonates endorses cup rather than bottle feeding as a temporary substitute for breastfeeding.

## Background

Health policies worldwide have prioritized the promotion, protection and support of breastfeeding, as a key strategy for reducing child mortality and improving the quality of health in the population [1]. One global policy is the Baby-friendly Hospital Initiative (BFHI), originally announced in 1991–1992 by the World Health Organization (WHO) and the United Nations Children's Fund (UNICEF), revised in 2009, has established the Ten Steps to Successful Breastfeeding [2]. One of the benefits of breastfeeding is the promotion of craniofacial growth and development. Exclusive breastfeeding provides oral maturation by stimulating muscle tone and the harmonic development of the stomatognathic system [3-9].

Step 9 of the BFHI affirms that newborns should not be fed with bottles/artificial teats mainly because they negatively interfere with breastfeeding demand and so determine the failure of breastfeeding [2]. Nevertheless, in the neonatal period, certain situations such as maternal disease or emotional disturbances, as well as anatomical changes in the breast, can interfere with milk production and temporarily hinder exclusive breastfeeding [10]. In such circumstances, alternative sources, such as a cup, should be recommended until exclusive breastfeeding can be restored [1,2,11-13].

Cup feeding as a transitional method prior to breastfeeding has several advantages according to a revision by an expert group [11], such as improving the likelihood of late preterm infants being exclusively breastfed after discharge without increasing their hospital stay [14]. In a longitudinal study with full term infants, cup-feeding was found to be better than bottlefeeding regarding infant sucking behavior and maternal milk supply [15]. However, a systematic review based on few trials concluded that cup feeding would not be so beneficial for preterm infants because it delayed hospital discharge and was associated with staff and parental dissatisfaction, even though it increased the likelihood of exclusive breastfeeding at discharge [16]. In contrast, bottle feeding has been contraindicated because it is associated with nipple confusion and early weaning and could be linked to the onset of various conditions including gastrointestinal, ear, and respiratory infections, parafunctional oral habits, changes in sucking, swallowing, chewing and speech, and malocclusions [7,11,13,17]. There are evidences that bottle feeding leads to unsuccessful breastfeeding in full term and preterm infants [2,11,16].

During breastfeeding, suction primarily depends on the orofacial muscles –the masseter, temporalis, medial and lateral petrigoideos and suprahyoid muscles–working together in the extraction of breast milk [9]. The masseter is a powerful muscle which is responsible for the movements of mandibular protrusion, elevation and retrusion [3,7-9,18-20]. As there is greater involvement of the masseter muscle, to the detriment of the other muscles involved, while sucking to the breast [18,21,22], masseter muscle activity during breastfeeding could be considered a standard with which to compare other alternative methods of feeding in the neonatal period.

Due to the anatomical configuration and location of the masseter muscle on the surface of the facial region, its activity can be measured using surface electromyography (EMG), a safe and non-invasive method [23,24] introduced into research in the last decade which allows for objective quantification of muscle energy [24,25]. It is a safe effective method for evaluating the orofacial muscles of preterm [12] or term infants [18,21,22], children and adults [25,26] during feeding.

Various studies have assessed muscle activity by means of surface electromyography during breastfeeding and other feeding methods in the first year of life, and have concluded that the masseter is more active in breastfed than in bottlefed or cup fed infants [18,21,22,27,28]. However, little is known about muscle activity during breastfeeding in newborns. They do not have any established pattern of muscle activity during suction while older infants already have a predominant pattern depending on the type of feeding they have received.

The aim of this study was to analyze with the use of EMG the electrical activity of the masseter muscle during suction in term newborns, by comparing breast, bottle and cup feeding. The hypothesis was that the masseter presents higher electrical activity during breastfeeding when compared to the other two methods. This knowledge is crucial for defining alternative methods (versus bottle feeding) which promote a level of muscle activity similar to that seen in breastfeeding, therefore potentially contributing to support of mothers’ establishment and continuation of breastfeeding.

## Methods

### Study design

This observational, crosssectional, analytical study was carried out in the rooming-in ward of a mother and child referral hospital (Hospital Materno-Infantil “HMI”) in Goiania, capital of the state of Goias, a city of over a million inhabitants, located in central Brazil. The HMI is a public Baby Friendly accredited hospital (since 1999) where rooming-in is provided in eight wards totaling 40 beds; newborns stay with their mothers until discharge. This study was approved by the HMI Research Ethics Board (protocol #21/2011). The principal researcher invited mothers individually to allow their newborns participate in the study. After explaining the aim, procedures, benefits and risks of the study, the mothers were asked to sign the consent form.

### Participants

Participants were included according to the criteria: clinically stable full-term newborns presenting with 37–42 weeks of gestational age and birth weight of 2000 to 4000 g. Exclusion criteria were craniofacial deformities, difficulty in sucking and newborns whose mothers refused to participate in the study.

The sample was calculated on the basis of the results of a pilot study with 15 newborns (5 per group), where there was a minimum difference of 37.4% in masseter activity when breast and cup feeding were compared. So 27 newborns were needed to reject the null hypothesis with 80% power and the possibility of a type II error of 5%.

### Procedures

The principal researcher assessed one newborn a day. There was no research intervention in the type of feeding that newborns were using, which followed the hospital protocol: breastfeeding was recommended for infants from mothers with satisfactory milk supply; cup feeding, as a transitional method for infants from mothers with inadequate milk supply; bottle feeding, as a permanent method for infants whose mothers were not allowed to breastfeed because of a systemic disease (e.g. AIDS). That is, infants were not exposed to methods other than those of their group before EMG assessment. Non-breastfed newborns received breast milk from the HMI Milk Bank. EMG assessment was determined by the time when each infant showed interest in sucking.

**Figure 1:**
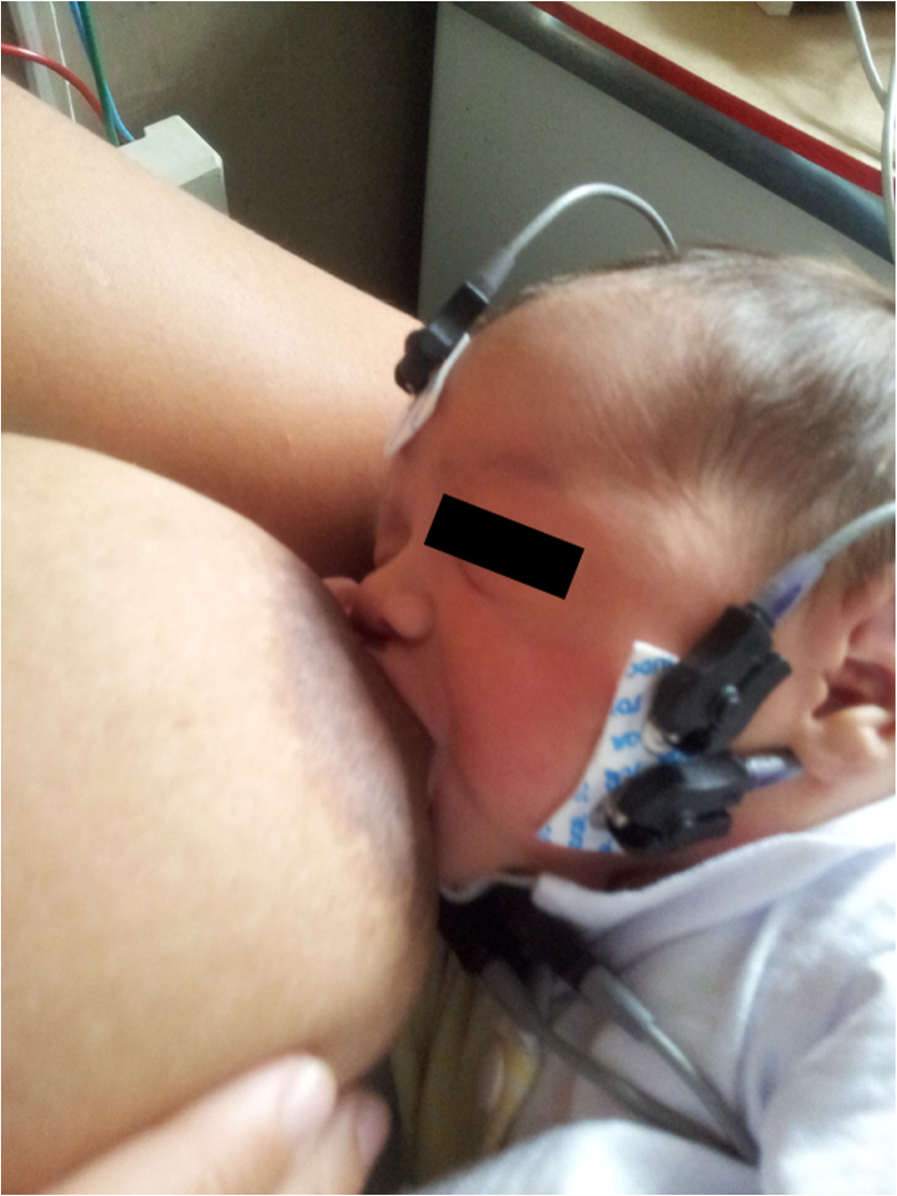
**Breastfeeding.** Breastfeeding with electrodes attached on the left hemiface during suctioning (mother gave specific consent to publish this figure).

**Figure 2:**
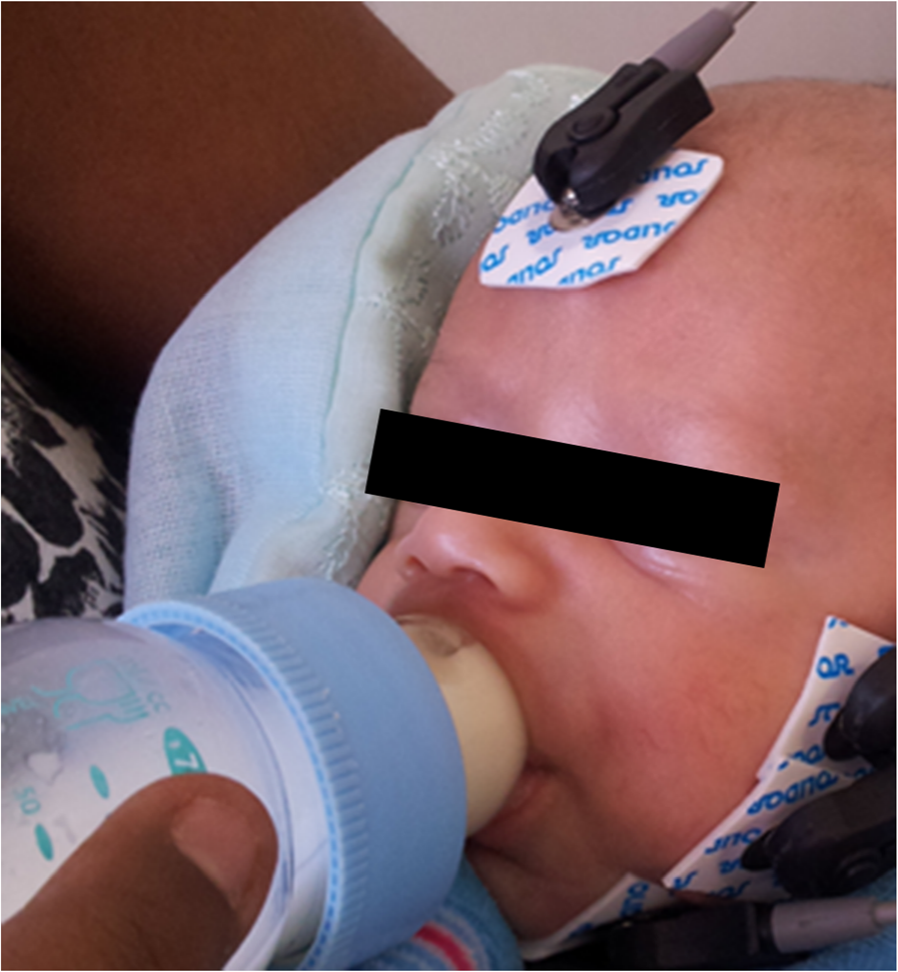
**Bottle feeding.** Bottle feeding with electrodes attached on the left hemiface during suctioning (mother gave specific consent to publish this figure).

**Figure 3:**
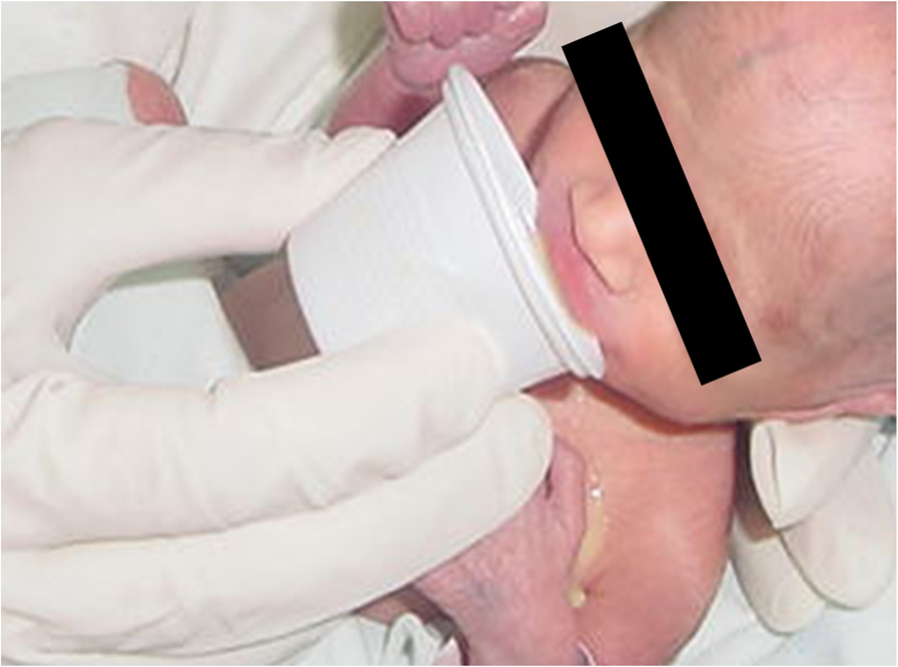
**Cup feeding.** Cup feeding just after electrodes removal (mother gave specific consent to publish this figure).

Consent forms and demographic data were completed before the infant’s feeding. Then, while the newborn was either suckling its mother’s breast (Figure 1), a bottle with orthodontic nipple (Figure 2) or cup (Figure 3), masseter electromyographic activity was assessed during the first 5 minutes of suckling. The newborns facial skin was cleaned with 70% alcohol before attaching the electrodes. Disposable pre-gelled electrodes of unipolar surface, silver-silver chloride (Ag-AgCl) composition (Solidor® MSGST-06, Medico Electrodes International, Uttar Pradesh, India) were first reduced in size to 10 mm, to fit the newborn’s cheek [29]. Then, the first reference electrode was applied to the glabella region. This was followed by placing the other two electrodes, with a minimum of 10 mm apart, on the left hemiface, over the masseter belly, in a bipolar configuration, arranged longitudinally along the muscle fibers, in accordance with the SENIAM (Surface electromyography for the Non-Invasive Assessment of Muscles) guidelines [30] (Figures 1 and 2).

The EMG was performed with a Miotool 200 (Miotec Equipamentos Biomedicos Ltda - ME, Porto Alegre, Brazil) four-channel device coupled to a notebook, set to a nominated gain of 1000 and filters of 20 Hz (low pass) and 500 Hz (high pass). The EMG device also included sensors for connection with claws, reference cable (earth) and calibrator (Miotec®) (SENIAM) [29].

### EMG signal analysis

Masseter electrical activity was analyzed with the Miograph 2.0 (MIOTEC®, São Paulo, Brasil) software. Digitalized signals were converted to Root Mean Square (RMS), which is the result of the square root of the mean squared amplitudes for the instantly recorded EMG signals, expressed in microvolts (μV).

For each newborn, means (μV) were transformed into a percentage of the reference value. The formula to calculate the percentage, according to the International Society of Eletrophysiology and Kinesiology – ISEK [25] is (X/Y) times 100, where X = mean of the electrical muscle activity (EMA) in the specified task (μV); and Y = reference value corresponding to the mean of peak AEM (μV). The EMG data were normalized by maximum peak [31] and the highest value for masseter activity for 3 seconds was obtained. Maximum peak was then 100% of activity and the mean of activity for 3 seconds was “X” [31].

### Statistical analyses

The dependent variable was the RMS value observed in the three feeding methods, which followed a normal distribution (Kolmogorov-Smirnov *P* = 0.394). The independent variables were gestational age, birth weight (grams), Apgar score and age (days) at the moment of the EMG examination. After descriptive analyses, the association between the RMS and other independent variables was tested by one-factor analysis of variance (ANOVA) and the Bonferroni test for post-hoc comparisons. For the linear regression analysis, the forward stepwise method was considered the best model to predict the electrical activity of the masseter muscle (RMS). Significance level was set at 5%. Analyses were performed using IBM SPSS software v. 19.

## Results

The participants were 81 term neonates, 1 to 28 days of life. Of these, 50.6% (n = 41) were males and 49.4% (n = 40) females, all featuring an adequate non-nutritive sucking pattern. There were 27 neonates per group; there was no difference between the groups in terms of infant characteristics (Table 1).

**Table 1:** Infants’ characteristics

**Variables**	**Feeding method mean (range)**			***P*** *
	**Breastfeeding (n = 27)**	**Bottle-feeding (n = 27)**	**Cup-feeding (n = 27)**	
**Gestational age**	38.7 (37.0-41.5)	38.8 (37.0-41.0)	38.9 (37.0-41.6)	0.773
**Birth weight (grams)**	3127.2 (2285.0-4499.0)	3089.6 (2300.0-4100.0)	3158.5 (2355.0-4045.0)	0.884
**Apgar**	8.9 (3.0-10.0)	9.0 (7.0-10.0)	9.4 (7.0-10.0)	0.265
**Age at electromiographic exam**				
**- Chronological (days)**	4.9 (2.0-15.0)	5.6 (1.0-28.0)	4.2 (2.0-11.0)	0.539
**- Postmenstrual (weeks)**	39.4 (37.3-42.2)	39.6 (37.3-41.3)	39.5 (37.3-41.9)	0.503

Association between infants’ characteristics and feeding method.

*ANOVA.

**Figure 4:**
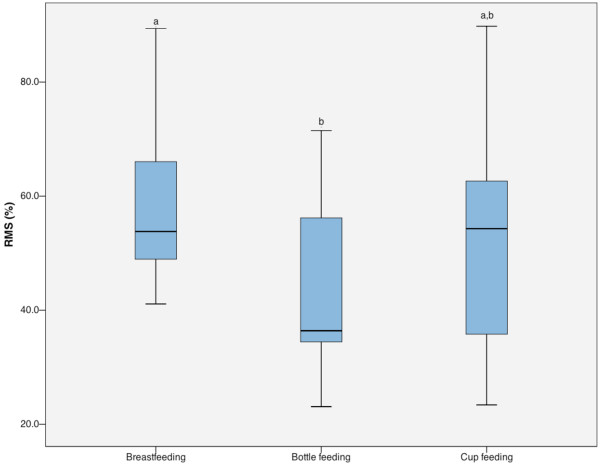
**Masseter activity according to the type of feeding.** Masseter activity in percentage of Root Mean Square (RMS), assessed during newborn feeding in breast, bottle or cup. Different letters (a,b) indicate statistically significant differences (*P* = 0.003) between groups.

The EMG assessment showed that the percentage of RMS values for masseter activity were significantly lower in the bottle feeding group (mean 44.2%, range 23.1-71.5) than in the breast feeding (mean 58.3%, range 41.1-89.4) (*P* = 0.003, ANOVA), while the cup feeding RMS values (mean 52.5%, range 23.4-89.8) did not differ from the aforementioned methods (Figure 4). Comparison between bottle and cup feeding showed the power of 46.6% to detect a statistically significant difference.

The spreadsheet was optimized to increase model fitness in the multiple regression analysis by excluding 4 cases with outliers in RMS values, and dicotomizing the variable “feeding method” into breast versus bottle/cup. Initially all independent variables were included in the model, but only two remained in the final model (Table 2): birth weight showed a positive relationship with RMS and bottle/cup-feeding a negative relationship. For every gram of birth weight increase, RMS increased by 0.010 units. Bottle/cup feeding reduced RMS by 10.392 units, when compared to breastfeeding. The coefficient of determination (R^2^), although statistically significant, was low: the model explained 23.9% of the variation in RMS.

**Table 2:** Regression model for masseter activity

**Variables in model**	**Odds ratio (OR)**	***P***	**95% confidence interval for OR**	
			**Upper bound**	**Lower bound**
Birth weight (grams)	0.010	0.001	0.004	0.016
Bottle-feeding	-10.392	0.002	-16.741	-4.042

Final regression model for variables predicting masseter muscle activity in electromiography (EMG) of term infants (in percentage of Root Mean Square).

R^2^ = 0.239 (*P* = 0.002).

## Discussion

The outcome of the study partially confirmed our hypothesis, because it showed that the level of masseter electrical activity was higher during breastfeeding than that observed during bottle feeding, but that of cup feeding was similar to both breast and bottle feeding. In addition, RMS was significantly predicted by birth weight as well as by bottle and cup feeding.

One of the strengths of this study is that infants were fed in response to their behavioral signs of interest in suckling, despite their group (breast, bottle or cup feeding). This attittude facilitates the establishment of oral feeding and have other favorable impact on infant’s growth and discharge, if compared with feeding at intervals planned by the health personnel [11]. Probably, if we had done the EMG assessment at scheduled intervals, the masseter muscle activity could have showed different sucking patterns.

During breastfeeding, there is intense jaw movement, especially mandibular elevation and protrusion, and the masseter muscle is mainly responsible for the execution of these movements [3,7-9,18-20]. It is believed that the muscular activity which occurs during breastfeeding is vital for adequate harmonious craniofacial growth [3,4,6,7,9].

The similarity between masseter muscle activity comparing exclusive breastfeeding with breastfeeding/cup was also reported in another study with older infants [18]. Although there are few studies on electromyography during cup feeding, it is believed that the movements of the jaw and tongue observed during cup feeding are similar to those performed to the breast [32]. The mechanism used by the baby to remove milk from the cup is “sucking/licking” [32], that is, the tongue movement is accentuated, with a predominance of a vertical motion of the jaw (mouth closing). As movements during breastfeeding are more complex and include jaw elevation, lowering, protrusion and retrusion [3,7-9,18-20], it is believed that the cup does not facilitate the mechanism for breastfeeding [33]. Nevertheless, our results support the recommendation of cup feeding when the newborn needs an alternative feeding method, as it provides the infant with an opportunity for developing the muscles involved in suction [9,32]. The positive impact of cup feeding has also been reported in studies where there is a higher prevalence of breastfeeding after hospital discharge in infants who used the cup for supplementation in neonatal units [17,32,34].

Our results are consistent with other EMG results which showed significantly reduced activity of the masseter muscle in bottle-feeding when compared to breastfeeding [18,21,22]. Interestingly, in this study cup/bottle-feeding significantly decreased the RMS percentage in the regression model by a mean of 10 percentage points. In bottle-feeding, the masseter, temporalis, pterygoid, tongue and lips operate in hypofunction, while the mentalis and buccinator are in hyperactivity [18]. This increased activity of the buccinator muscle reduces jaw movements, promotes tongue retraction and increases the chances of tongue hypoactivity or hyperactivity, depending on the type of suction performed [9,18]. This could affect the development of the masticatory function, and lead to possible chewing and swallowing disorders [7,8,19,27,34].

This study found that the activity of the masseter muscle during cup feeding was higher than that of bottle-feeding, although not statistically significant. On the contrary, the activity of the masseter muscle in 20 breastfed older infants during cup feeding was significantly higher when compared to sucking a bottle in 20 infants with routinely mixed feeding (breast plus bottle-feeding) [18]. In that study [18], the infants who only had cup feeding for the EMG had been routinely breastfed and this could have benefited their muscle activity. In the present study, infants did not receive mixed feeding. Also, in our study the power to observe a statistically significant difference between cup and bottle feeding was lower than 80%, so further studies should be undertaken with larger sample sizes.

In addition, the regression model in our study showed that every gram increase in an infant’s birth weight would increase RMS by 0.010 percentage points. Although this change might not be clinically relevant, it should be emphasized that electrical muscle activity in infants depends on neuromuscular maturity, craniofacial growth and clinical condition [28]. In this study, we sought to control the above-mentioned characteristcs in the groups included in this study, in order to reduce possible individual interferences in the electrical activity of the masseter muscle. This explains why no differences in masseter activity were found with regard to gestational age, birth weight, days of life on the day of the test or Apgar when the three feeding groups were compared.

Although the sample size was the major limitation of this study, other EMG studies which compared feeding methods in children worked with samples varying from 12 to 20 infants per group. In addition, comparisons are difficult when different infant ages, muscle and feeding methods are involved [18,21,22,27,28]. So, further EMG studies analysing sucking in newborns are needed to establish the pattern of muscle activity involved in each different feeding method, to help choose the method which best fosters craniofacial development in a transitory period when breastfeeding is not possible.

The clinical impact of the present outcomes can be speculated, if we consider the literature already published. Overall this study showed that the superiority of masseter activity during breastfeeding as compared to bottle feeding can be seen in the first week of life in term infants. Then, Step 9 from the BFHI [2,11] discouraging bottle-feeding of infants whose mother intends to breastfeed, at least during the establishment of breastfeeding, is confirmed from the viewpoint of muscle development. Also, the controversy about the benefits and disadvantages of cup feeding can be debated [11-16]. The present findings on masseter activity support the understanding that the cup-feeding is somewhat more similar to breastfeeding in healthy term neonates. As the setting of this study is a Baby Friendly accredited hospital, probably the staff does not find major issues in training the mother to feed the infant with a cup, as other reports suggest [16].

## Conclusions

Masseter activity was significantly higher in breastfed than in bottle-fed newborns, who presented the lowest RMS values. The level of masseter activity during cup-feeding was between that of breast and bottle feeding, and did not significantly differ from either group. Cup/bottle feeding significantly predicted a decrease in RMS, when compared to breastfeeding.

In view of the intense debate about the best temporary substitute for breastfeeding, this study in healthy full term neonates endorses cup rather than bottle feeding.

## Competing interests

The authors declare that they have no competing interests.

## Authors’ contributions

ECLF: Conception and design of study; acquisition, analysis and interpretation of data; drafting of article. CBS: Conception and design of study; analysis and interpretation of data; drafting of article. LCA:Analysis and interpretation of surface electromyography data, review of manuscript; LRC: Conception and design of study; acquisition, analysis and interpretation of data; drafting of article. All authors contributed in revision and agreed on the final manuscript.

## Pre-publication history

The pre-publication history for this paper can be accessed here:

http://www.biomedcentral.com/1471-2393/14/154/prepub
